# Arthroscopic-Assisted Lower Trapezius Tendon Transfer for Irreparable Posterosuperior Rotator Cuff Tears Using a Quadricipital Tendon Allograft and a Double-Row Construct

**DOI:** 10.1016/j.eats.2024.103188

**Published:** 2024-08-23

**Authors:** Caio Santos Checchia, Renato Miyadahira, Eduardo Angeli Malavolta, Jorge Henrique Assunção, Sergio Luiz Checchia, Alberto de Castro Pochini, Carlos Vicente Andreoli, Benno Ejnisman

**Affiliations:** a Hospital das Clínicas da Faculdade de Medicina da Universidade de São Paulo, Sao Paulo, Brazil; b Hospital Sírio-Libanês, Sao Paulo, Brazil; c Hospital do Coração, Sao Paulo, Brazil; d Diagnóstico das Américas Sociedade Anônima/Hospital 9 de Julho, Sao Paulo, Brazil; e Centro de Traumato-Ortopedia do Esporte, Universidade Federal de São Paulo, Sao Paulo, Brazil

## Abstract

Lower trapezius transfer is a joint salvage treatment option for irreparable posterosuperior rotator cuff tears in relatively young patients. It requires the use of a tendinous graft to bridge the gap between the lower trapezius and the greater tuberosity. Inlay fixation of hamstring autografts and onlay single-row fixation of Achilles allografts have been described in the literature. This technical note describes an arthroscopic-assisted lower trapezius transfer using a quadricipital tendon allograft fixed with an onlay knotless double-row construct.

Surgery is often necessary for irreparable posterosuperior rotator cuff tears. For young, active patients, options range from arthroscopic debridement and partial repair to superior capsular reconstruction and tendon transfers.^1^ The most common transfer is that of the latissimus dorsi tendon (LDT),^1^ with variable reported outcomes. Although some have shown long-lasting improvement,^2^ clinical failures up to 36% have been reported.^3^

In 2016, Elhassan et al.^4^ described the lower trapezius tendon transfer (LTT), which offers a synergistic transfer with greater excursion and a force vector more closely aligned with the infraspinatus.^5^ Although long-term results are lacking, early to mid-term results demonstrate considerable improvement compared with LDTs.^6^ Nonetheless, given the medial insertion of the lower trapezius tendon in the scapular spine, a graft augmentation is invariably necessary to bridge the gap.^7^^,^^8^ For this purpose, either hamstring autografts or Achilles tendon allografts have been used.^5^

Compared with an autograft, an allograft has the advantage of no donor site morbidity and the possibility of being a larger and thicker tendon, which may be important for long-term integrity and its concomitant role as a subacromial spacer.^9^ However, the availability of allografts depends on donors, which makes supply and widespread application unpredictable. Ideally, allografts other than Achilles tendons could be used.

This study describes an arthroscopic-assisted LTT to the greater tuberosity, augmented with a quadricipital tendon allograft and fixed in a double-row manner.

## Surgical Technique

In our practice, only patients fulfilling the following 8 criteria are indicated for surgery: (1) absence of “true” pseudoparesis (i.e., active forward flexion <90° with anterosuperior escape of the humeral head); (2) age <70 years; (3) involvement of at least supraspinatus and infraspinatus tendons; (4) fatty infiltration Goutallier grade ≥III; (5) no subscapularis tears Lafosse grade ≥IV; (6) cuff tear arthropathy Hamada grade ≤II; (7) failure of conservative treatment after at least 4 months of uninterrupted physiotherapy; and (8) patient consent that there is no expectation of complete functional recovery after operation.

**Fig 1:**
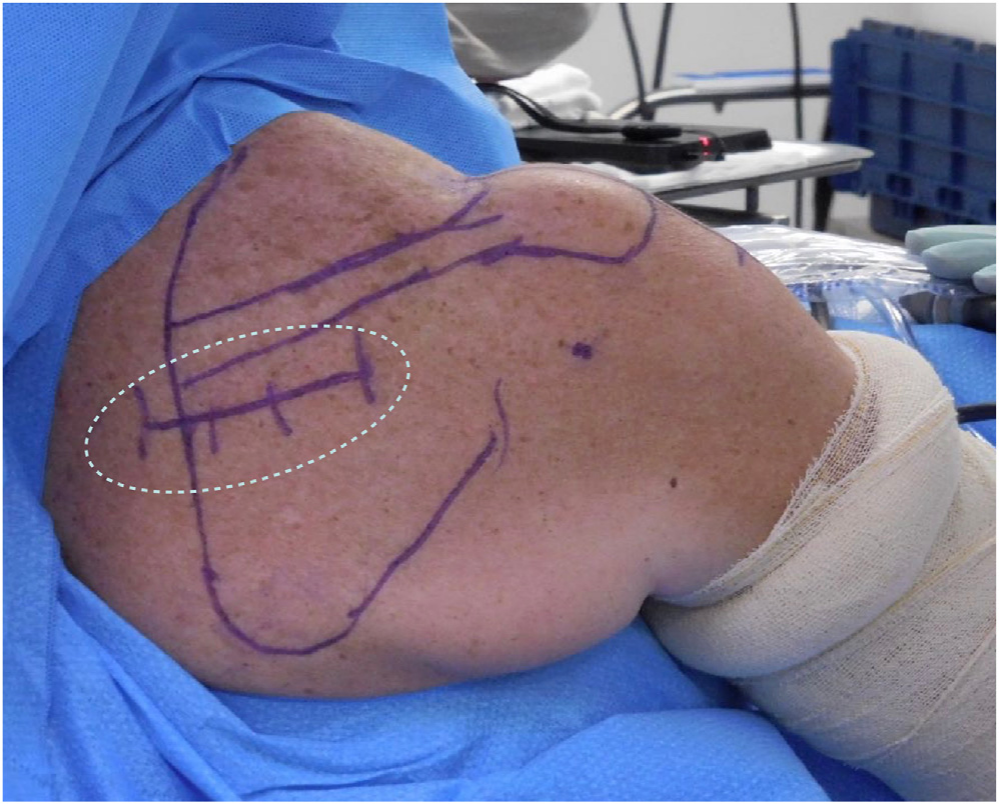
Right shoulder, posterior view. The patient is in the beach-chair position with the torso displaced as laterally as possible to expose the entire scapula. The site of the posterior incision (encircled by dashed line) is marked on the skin 2 cm below the medial border of the scapular spine.

After general anesthesia and interscalene brachial plexus block, the patient is placed in the beach-chair position with the torso displaced as laterally as possible to expose the entire scapula (Fig 1, Video 1).

**Fig 2:**
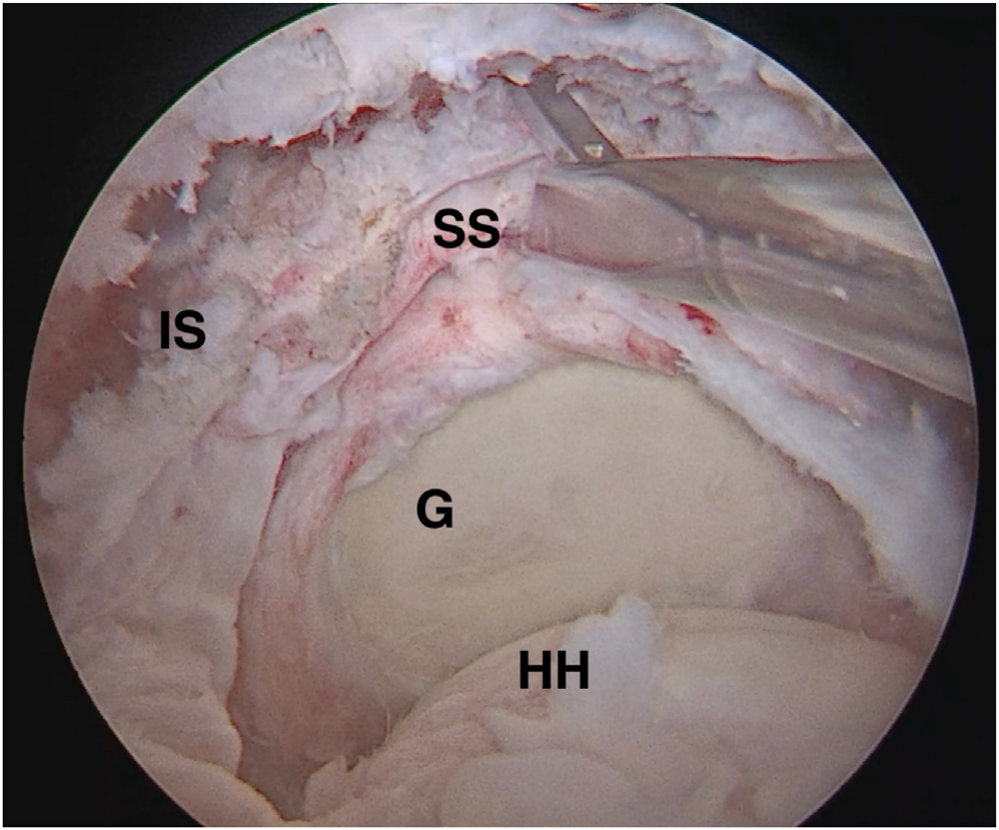
Right shoulder, view from lateral arthroscopic portal. Note the massive, retracted tear involving the posterosuperior rotator cuff. (G, glenoid; HH, humeral head; IS, infraspinatus tendon; SS, supraspinatus tendon.)

**Fig 3:**
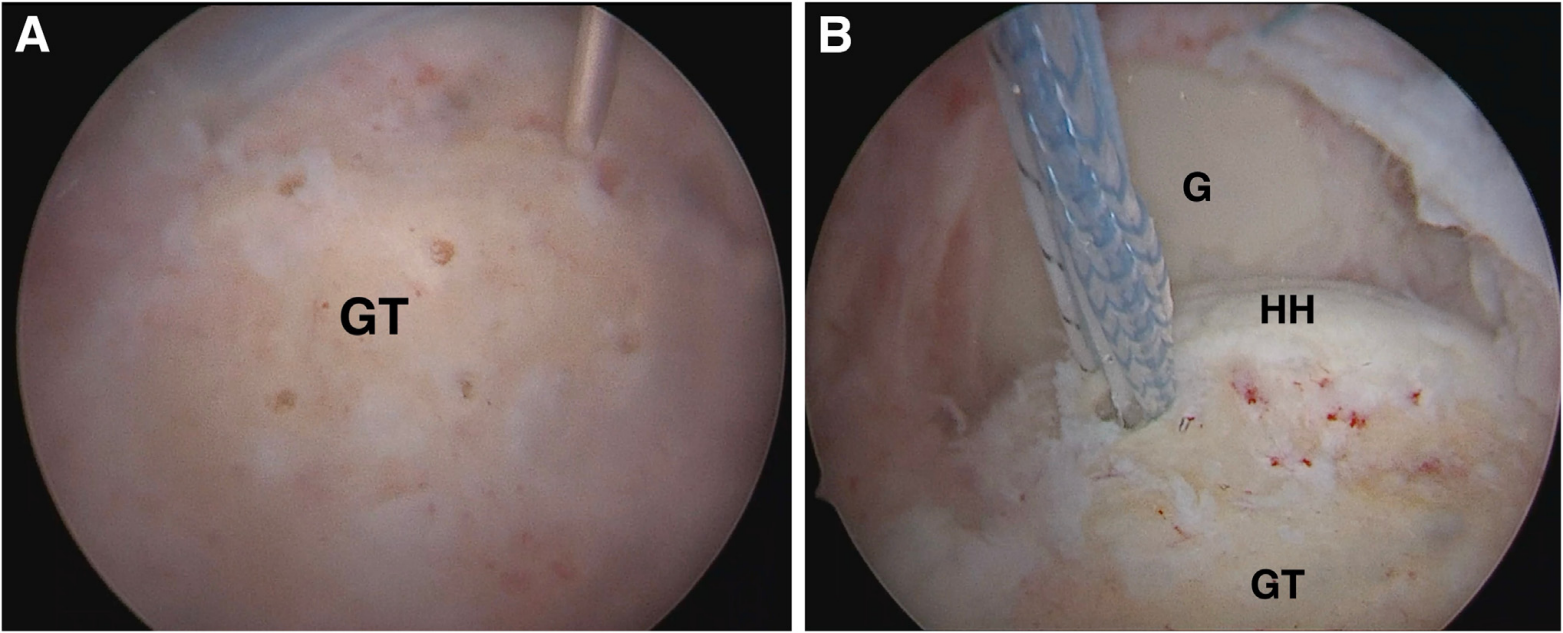
Right shoulder, view from lateral arthroscopic portal. (A) Marrow vents being perforated in the greater tuberosity using a 1.2-mm Kirschner wire with a motorized drill. (GT, greater tuberosity.) (B) A knotless 4.75-mm SwiveLock anchor (Arthrex), loaded with 2 limbs of a single Fibertape, is placed in the posteromedial aspect of the footprint, to be used later to fix the transfer. (G, glenoid; GT, greater tuberosity; HH, humeral head.)

Surgery begins arthroscopically. Whenever there is a long head of the biceps lesion, it is tenotomized. Concomitant subscapularis tendon tears are repaired in a standard fashion. No acromioplasty is performed. Rotator cuff irreparability is confirmed if a tension-free repair is not feasible (Fig 2), but a partial repair is done whenever possible. The footprint is prepared and measured, and marrow vents are drilled into the greater tuberosity (Fig 3A). A knotless 4.75-mm SwiveLock anchor (Arthrex, Naples, FL, USA), loaded with 2 limbs of a single Fibertape, is placed in the posteromedial aspect of the footprint for later use (Fig 3B) (Table 1 and Video 1).

**Table 1:** Tips and Tricks

1. The tendinous part of the allograft should be at least 15 cm long.
2. Perform any additional arthroscopic procedure before graft fixation, after which arthroscopic subacromial visualization becomes difficult.
3. Before transferring the graft into the subacromial space, fix the posteromedial anchor and retrieve its tapes to an anterior (or a Neviaser) portal, then drill marrow vents in the greater tuberosity.
4. The lower trapezius tendon is fragile. Make sure to reinforce its lateral border with a continuous suture using a strong braided wire before the Pulvertaft-like weave.
5. During surgery, the use of an articulated arm holder is preferred because the shoulder must be kept in maximal external rotation and approximately 30° of abduction during the Pulvertaft-like weaving of the graft into the lower trapezius and during skin closure.
6. Immobilize the shoulder in external rotation and abduction with a triangular abduction sling or a rigid shoulder orthosis.

**Fig 4:**
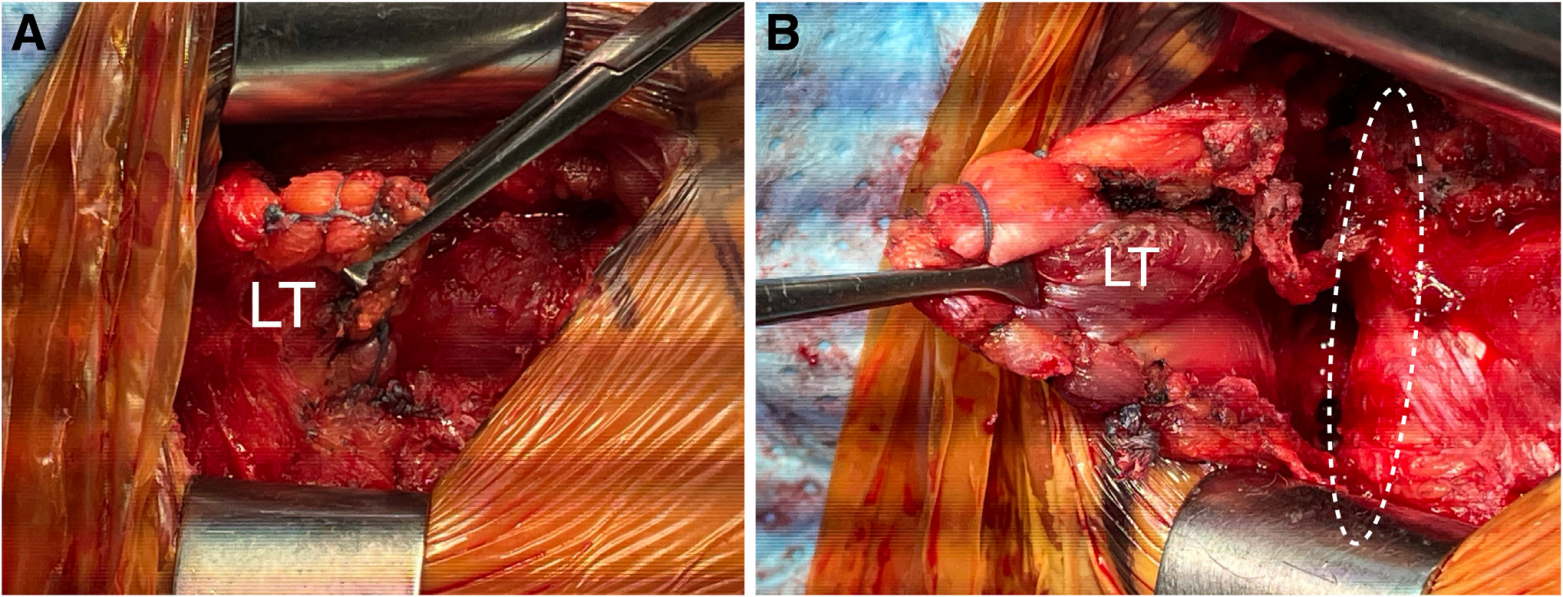
Right shoulder, posterior view. Close-up pictures of the posterior open approach, after the lower trapezius tendon (which is held with a clamp) had been detached from the scapula spine and mobilized freely. Note that the detached lateral portion of tendon was reinforced with a continuous suture using a No. 2 Fiberwire. (A) The detached tendon is being pulled laterally. (LT, lower trapezius.) (B) The detached tendon is mobilized medially. Close attention is paid to the accessory nerve, which can be palpated running anterior to the lower trapezius and approximately 2 cm medial to the scapula. The dashed line encircles the medial scapular border. (LT, lower trapezius.)

A 5- to 7-cm-long horizontal incision is made 2 cm below the medial part of the scapula spine (Fig 1). The lower trapezius tendon is detached from the scapula spine and freely mobilized (Fig 4 A and B). Close attention is paid to the accessory nerve, which runs anterior to the lower trapezius and approximately 2 cm medial to the scapula (Fig 4B). The detached lateral portion of tendon is reinforced with a continuous suture using a No. 2 Fiberwire (Fig 4 A and B) (Table 1 and Video 1)

**Fig 5:**
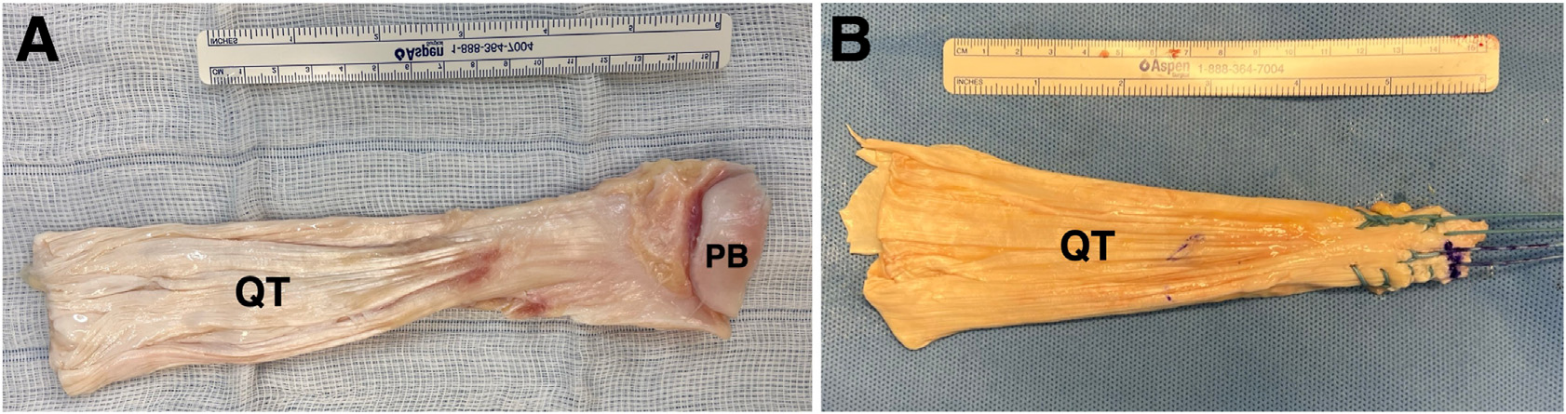
Picture of a quadricipital tendon (QT) allograft before (A) and after (B) preparation (over the surgical table). Any remaining patellar bone (PB) is removed, and the distal tendon is cut to the same width of the greater tuberosity footprint. Two Krackow sutures are made with No. 5 Fiberwires, leaving 4 limbs of wire.

The allograft is prepared over the table. Any remaining patellar bone is removed, and the distal tendon is cut to the same width of the greater tuberosity footprint. Two Krackow sutures are made with No. 5 Fiberwires (Arthrex), leaving 4 limbs of wire (Fig 5 A and B) (Table 1 and Video 1).

**Fig 6:**
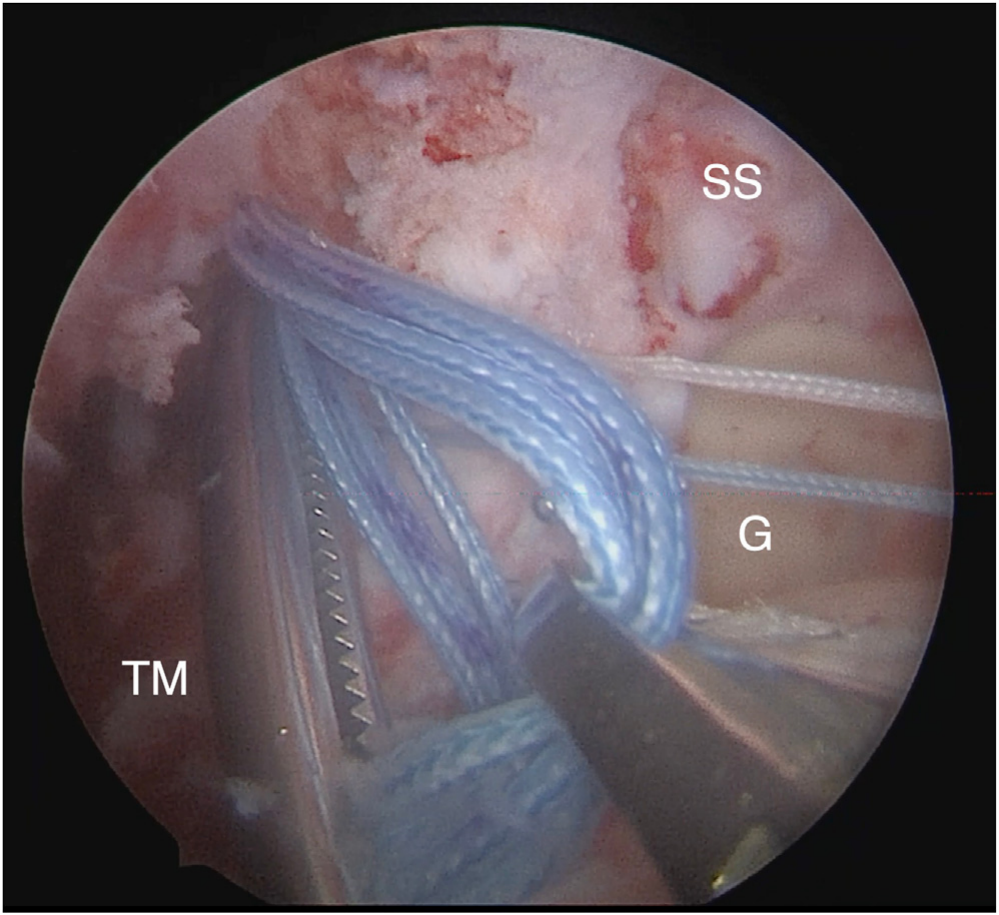
Right shoulder. View from lateral arthroscopic portal. The clamp on the left is passed from the posterior open approach into the subacromial space, holding the 4 limbs of wire from the 2 Krackow sutures (previously placed in the graft). The clamp on the right is placed through the anterolateral arthroscopic portal to retrieve the wires. (G, glenoid; SS, supraspinatus; TM, teres minor.)

To connect the posterior approach to the subacromial space, the interval between the infraspinatus and the posterior deltoid is dissected bluntly. A long clamp (holding the 4 limbs of wire) is then passed from the posterior into the subacromial space, and the wires are retrieved through the anterolateral arthroscopic portal (Fig 6) (Table 1 and Video 1).

**Fig 7:**
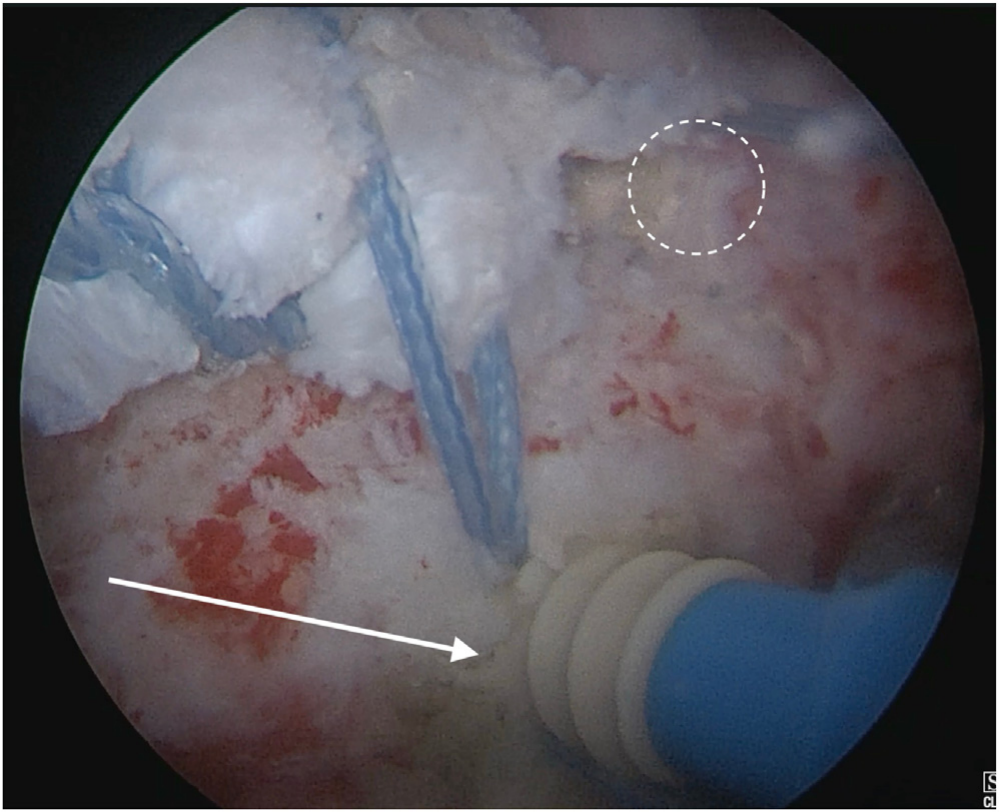
Right shoulder. View from lateral arthroscopic portal. A knotless, anterolateral 5.5-mm SwiveLock anchor (arrow) is placed to fix the 2 limbs of wire from the lateral Krackow suture. An identical anteromedial anchor had previously been fixed (dashed circle).

**Fig 8:**
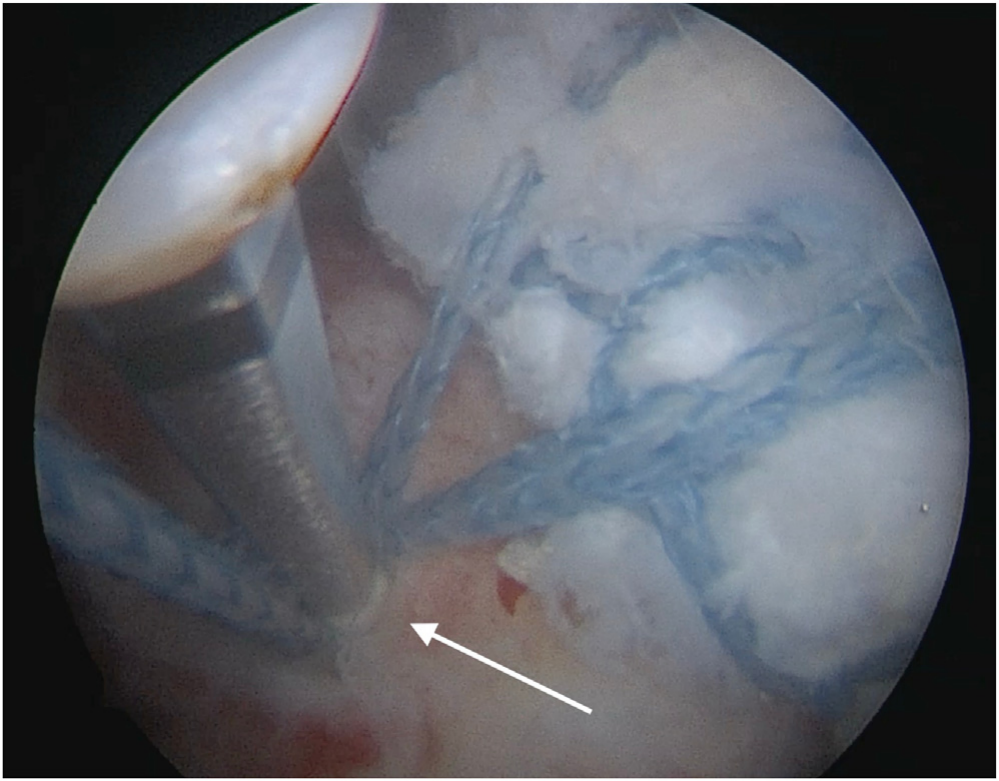
Right shoulder. View from lateral arthroscopic portal. Both limbs of Fibertape from the previous posteromedial anchor are being tensioned over the allograft and linked to a posterolateral 5.5-mm SwiveLock anchor (arrow) to compress the graft to the greater tuberosity.

The transfer is fixed with a double-row construct. Both limbs from the medial Krackow are fixed to a knotless anteromedial 5.5-mm SwiveLock anchor, and another identical anchor is placed anterolaterally to fix the 2 lateral limbs (Fig 7). Both anchors are placed just posterior to the bicipital groove, creating the anterior row of anchors. The second row is more posterior. Both limbs of Fibertape from the previous posterolateral anchor are retrieved, tensioned over the allograft, and linked to a posterolateral 5.5-mm SwiveLock anchor, compressing the graft to the greater tuberosity (Fig 8).

**Fig 9:**
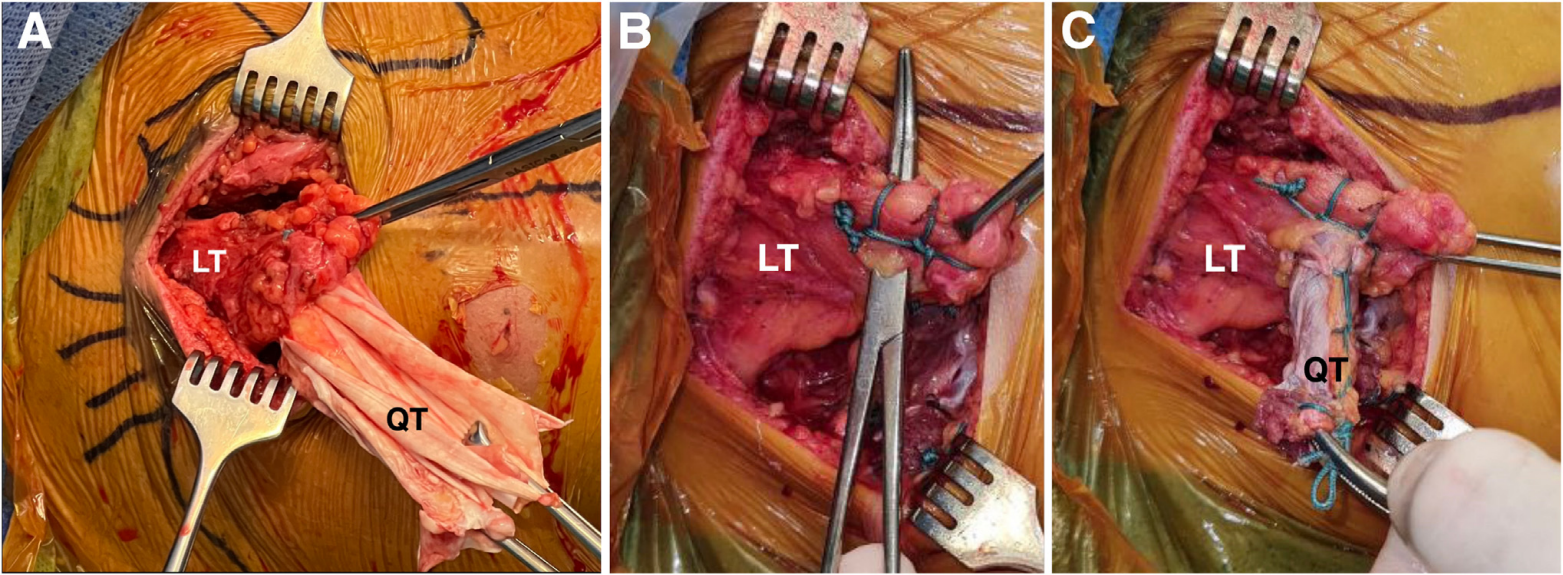
Right shoulder, posterior view. Close-up pictures of the posterior open approach. (A) LT, lower trapezius tendon; QT, quadricipital tendon allograft. (B) A clamp is used to perforate the lower trapezius tendon (LT). (C) The medial part of the graft (QT) is passed through the lower trapezius tendon (LT) to create a Pulvertaft-like weaving of both structures.

The shoulder is then placed in maximal external rotation and approximately 30° of abduction. A Pulvertaft-like weaving of the graft into the lower trapezius is performed and secured with multiple sutures (Fig 9 A-C). Finally, the shoulder is gently mobilized for inspection through the medial (open) and lateral (arthroscopic) approaches (Table 1 and Video 1).

**Fig 10:**
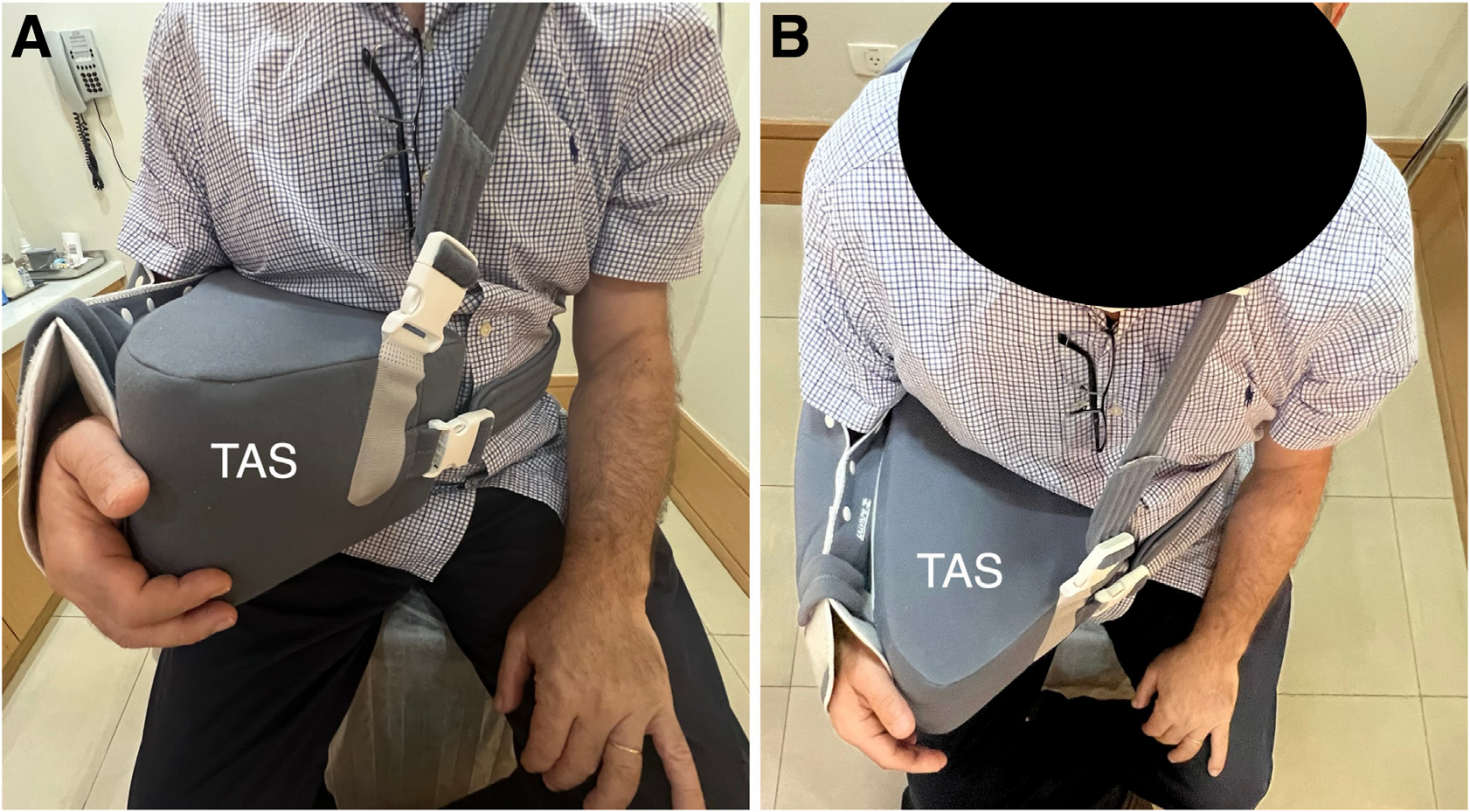
Anterior view. The right shoulder is immobilized in a triangular abduction sling (TAS) for 8 weeks after surgery. Postoperative photograph of a patient from anterior (A) and superior (B) views.

Postoperatively, the shoulder is immobilized in a sling with a triangular abduction pillow (Fig 10) for 8 weeks, after which rehabilitation with physical therapy begins to recover range of motion. Strengthening begins 4 months after surgery.

## Discussion

This surgical technique is a modification of Elhassan's original procedure,^4^ which uses an Achilles tendon allograft, fixed over the greater tuberosity with a single-row construct. The technique in this technical note aims to provide evidence for the use of an alternative allograft to the Achilles tendon and to illustrate an easy way to use a second (more posterior) row of anchors, and thus possibly achieve greater footprint coverage and tendon-to-bone compression (Table 2).

**Table 2:** Advantages and Disadvantages

Advantages
1. Use of a strong, thick graft with no donor site morbidity
2. Allograft availability may improve with an extra option for a strong tape-like tendinous graft
3. The thick onlay graft may also act as a subacromial spacer
4. The onlay double-row construct provides strong fixation without the disadvantages of inlay techniques
5. Optimal contact with the rotator cuff footprint
6. The double-row construct is easy and reproducible, without the need for arthroscopic suture passing or knot tying
Disadvantages
1. Allograft healing may be inferior compared with autologous tissue
2. Arthroscopic subacromial visualization is difficult after graft fixation
3. Use of additional knotless anchors increases costs

There is no consensus regarding the best type of graft to use as augmentation. To the best of our knowledge, only techniques involving tendons have been used, and the use of substitutes or alternative tissues has not been proposed.

Regarding graft length, given a reported mean gap of 10.9 cm (8.5-13.8 cm) between the native lower trapezius tendon and the lesser tuberosity, a graft of at least 15 cm long is required for adequate gap bridging and Pulvertaft-like weaving and fixation.^7^ We are unaware of any study investigating adequate graft width or thickness. It is reasonable to assume, however, that its width should allow coverage of the entire footprint to maximize the bone-tendon healing interface. Concerning its thickness, the graft should not be too thin (although a threshold has not been established) because it could compromise its strength and its potential role as a subacromial spacer. In addition, it should not be too thick to avoid subacromial impingement. Given these considerations, the ideal graft should have a tape-like shape (Table 2).

Although both tendinous autografts and allografts present viable options, there is no direct comparison for LTTs. In general, each comes with its advantages and disadvantages. Autografts generally exhibit lower long-term failure rates because of their inherent biological advantages in revascularization and integration with native tissue. However, early on, they demonstrate higher risk of rupture, likely because of the initial weakening during the remodeling phase.^10^ Although allografts offer the benefit of immediate structural strength, they have been associated with greater potential for long-term stretching and elongation.^10^ Additionally, the risk of immune response with allografts, although minimized through processing, could potentially compromise graft integrity.^11^ (Table 2).

To the best of our knowledge, in the setting of LTTs, the only allografts used were those of Achilles and posterior tibialis tendons. The latter, however, was used in only 1 study,^12^ and it was chosen specifically because it is a thinner tendon because the technique involved simultaneous LTT transfer and superior capsular reconstruction, so a larger Achilles tendon would make arthroscopic visualization difficult.^12^

There is still doubt regarding the best place for transfer fixation. There is no comparison of onlay (over the greater tuberosity) versus inlay (intraosseous) fixations in the setting of LTTs; therefore, part of our understanding comes from LDTs. In this scenario, tubularization of the tendon with transosseous fixation (cortical button) was reported to be strongest in vitro; however, it was shown to have a high failure rate in vivo, approaching 46%.^13^ This likely happens because the insertion of a tubularized tendon at a right angle into a bone results in the “killer turn effect,” which has been shown to increase tensile forces on the graft, leading to tunnel widening or tendon elongation and eventual failure.^13^ As such, onlay fixation has become increasingly popular.^5^

Regarding fixation, the majority of biomechanical studies have demonstrated significantly less gap formation during cyclic loading with a double-row compared with a single-row construct, as well as a larger tendon-bone interface,^14^^,^^15^ in the setting of rotator cuff repairs. Further investigation is warranted to explore these potential benefits in tendon transfers, but current evidence suggests that double-row techniques may promote more secure fixation and a more favorable healing environment. We propose that the ideal LTT transfer should be fixed with an onlay double-row construct, using a strong tape-like shaped tendon, such as an Achilles or, as shown in this paper, a quadricipital tendon graft (Table 2).

## Disclosures

All authors (C.S.C., R.M., E.A.M., J.H.A., S.L.C., A.d.C.P., C.V.A., B.E.) declare that they have no known competing financial interests or personal relationships that could have appeared to influence the work reported in this paper.

## Supplementary Data

Video 1 The patient is in the beach-chair position. This video demonstrates a modified arthroscopic-assisted lower trapezius transfer technique, using a quadricipital tendon allograft, which is fixed over the greater tuberosity (onlay) with a knotless double-row construct. This technique is performed in a right shoulder to treat an irreparable posterosuperior rotator cuff tear. The arthroscopic view is from the lateral portal with a 30^o^ scope, whereas the open approach is visualized posteriorly.

15
